# Gender Differences in Survival after Coronary Artery Bypass Grafting—13-Year Results from KROK Registry

**DOI:** 10.3390/jcm13144080

**Published:** 2024-07-12

**Authors:** Grzegorz Hirnle, Adrian Stankiewicz, Maciej Mitrosz, Sleiman Sebastian Aboul-Hassan, Marek Deja, Jan Rogowski, Romuald Cichoń, Lech Anisimowicz, Paweł Bugajski, Zdzisław Tobota, Bohdan Maruszewski, Tomasz Hrapkowicz

**Affiliations:** 1 Department of Cardiac Surgery, Medical University of Bialystok, 15-276 Bialystok, Poland; stankiewiczad@wp.pl (A.S.); mitrosz@gmail.com (M.M.); 2 Department of Cardiac Surgery and Interventional Cardiology, Faculty of Medicine and Medical Sciences, University of Zielona Gora, 65-417 Zielona Gora, Poland; s.aboul-hassan@inm.uz.zgora.pl; 3 Department of Cardiac Surgery, Upper-Silesian Medical Centre, Medical University of Silesia, 40-055 Katowice, Poland; mdeja@sum.edu.pl; 4 Department of Cardiac and Vascular Surgery, Medical University of Gdansk, 80-211 Gdansk, Poland; janrog@gumed.edu.pl; 5 Lower Silesian Center for Heart Diseases ‘Medinet’, Faculty of Medicine and Medical Sciences, University of Zielona Gora, 65-417 Zielona Gora, Poland; romuald.cichon@gmx.de; 6 Department of Cardiac Surgery, Dr Antoni Jurasz Memorial University Hospital, 85-094 Bydgoszcz, Poland; lech.anisimowicz@gmail.com; 7 Department of Cardiac Surgery, J. Struś Hospital, 61-285 Poznan, Poland; pawelbugajski@onet.eu; 8 Department of Paediatric Cardiothoracic Surgery, Children’s Memorial Health Institute, 01-210 Warszawa, Poland; ztobota@ecdb.pl.pl (Z.T.); bmar@ecdb.pl.pl (B.M.); 9 Department of Cardiac Surgery, Vascular Surgery and Transplantology, Silesian Centre for Heart Diseases, Medical University of Silesia, 41-800 Zabrze, Poland; t.hrapkowicz@sccs.pl

**Keywords:** coronary artery bypass grafting, gender differences, long-term survival

## Abstract

The influence of gender on both early and long-term outcomes of coronary artery bypass grafting (CABG) is not clearly defined. **Objectives:** This study aimed to assess the impact of gender on early and long-term mortality after CABG using data from the KROK Registry. **Methods:** All 133,973 adult patients who underwent CABG in Poland between 1 January 2009 and 31 December 2019 were included in the Polish National Registry of Cardiac Surgical Procedures (KROK Registry). The study enrolled 90,541 patients: 68,401 men (75.55%) and 22,140 women (24.45%) who met the inclusion criteria. Then, 30-day mortality, 1-year mortality, and long-term mortality rates were compared. **Results:** Advanced age, higher Canadian Cardiovascular Society (CCS) and New York Heart Association (NYHA) grade, diabetes, hypercholesterolemia, arterial hypertension, body mass index BMI > 35 kg/m^2^, and renal failure, before the propensity matching, were more frequently observed in women. Women more frequently underwent urgent surgery, including single and double graft surgery, and off-pump CABG (OPCAB) (*p* < 0.001). In propensity-matched groups, early mortality (30 days) was significantly higher in women (3.4% versus 2.8%, *p* < 0.001). The annual mortality remained higher in this group (6.6% versus 6.0%, *p* = 0.025). However, long-term mortality differed significantly between the groups and was higher in the male group (33.0% men versus 28.8% women, *p* < 0.001). **Conclusions:** There are no apparent differences in long-term mortality between the two sexes in the entire population. In propensity-matched patients, early mortality was lower for men, but the long-term survival was found to be better in women.

## 1. Introduction

Up to 30% of the CABG population are women [1,2]. Some studies report higher mortality and morbidity in women after surgical revascularization, which is explained by the smaller diameter of their coronary arteries and increased probability of incomplete revascularization [3,4,5]. Similar gender differences in survival were reported in patients undergoing percutaneous revascularization procedures [6].

Risk scales generally recognize the female gender as an independent risk factor, but do not consider biological differences and body habitus between men and women [7,8,9].

The influence of gender on early and long-term outcomes of CABG is not clearly defined. It is also not clear whether gender should influence the approach to surgical management of coronary artery disease. Usually, women receive a lower number of grafts and arterial revascularization [2]. However, a few studies have shown that operative technique (on-pump/off-pump) or arterial graft use did not play a role in the results of CABG with regard to gender [1,10].

There is a lot of discrepancy in the literature concerning the outcomes of women and men after CABG. To date, our study is the largest study with a long follow-up period presenting real-life data from a multicenter registry, which contributes significant information to this important topic.

The aim of this study was to evaluate the impact of gender on early and long-term mortality after CABG surgery.

### 1.1. Patients and Methods

The study used retrospective data collected from the KROK Registry (Polish National Registry of Cardiac Surgery Procedures, available at www.krok.csioz.gov.pl, accessed on 1 January 2020) from 2009 to 2019. This is a nationwide registry of all cardiac surgery procedures in Poland, linked to the National Health Fund, which tracks all deaths in the country since 2006. It is a joint initiative between the Polish Ministry of Health and Polish Society of Cardiothoracic Surgeons. All data were anonymized, and individual patient consents and ethics committee approval were not required. All cardiothoracic departments transfer their data to the National Centre for Healthcare Information System, which is under the supervision of the Ministry of Health [11]. Early mortality was defined as death due to any cause within 30 days of surgery. Follow-up data regarding the all-cause mortality of all patients were obtained from the National Health Fund, which is a nationwide, obligatory public health insurance institution in Poland.

### 1.2. Study Outcomes

The primary outcome of the study is the assessment of a long-term mortality assessed as all-cause mortality for 1 year and up to 13 years post-surgery.

The secondary outcomes include mortality within 30 days post-surgery. This mortality includes all causes of death, regardless of their origin, providing an evaluation of the immediate risk associated with the procedure.

### 1.3. The Postprocedural Complications and Their Definitions

- Neurological (new neurological deficit with persistent symptoms still present at the time of the hospital discharge).
- Respiratory (mechanical ventilation for more than 24 h, and/or pneumonia).
- Gastrointestinal (gastrointestinal bleeding, pancreatitis, cholecystitis, and/or mesenteric ischemia).
- Renal (renal replacement therapy).
- Surgical site infections (sternal, mediastinal or wound infection).
- Perioperative myocardial infarction according to the criteria used by the Society of Thoracic Surgeons adult cardiac surgery database.
- Mechanical circulatory support broadly defined as the use of any of the available options in this field.
- Intensive care unit (ICU) readmission (transfer to the ICU following a previous discharge from this unit, during the same hospital stay).

### 1.4. Study Population

**Figure 1:**
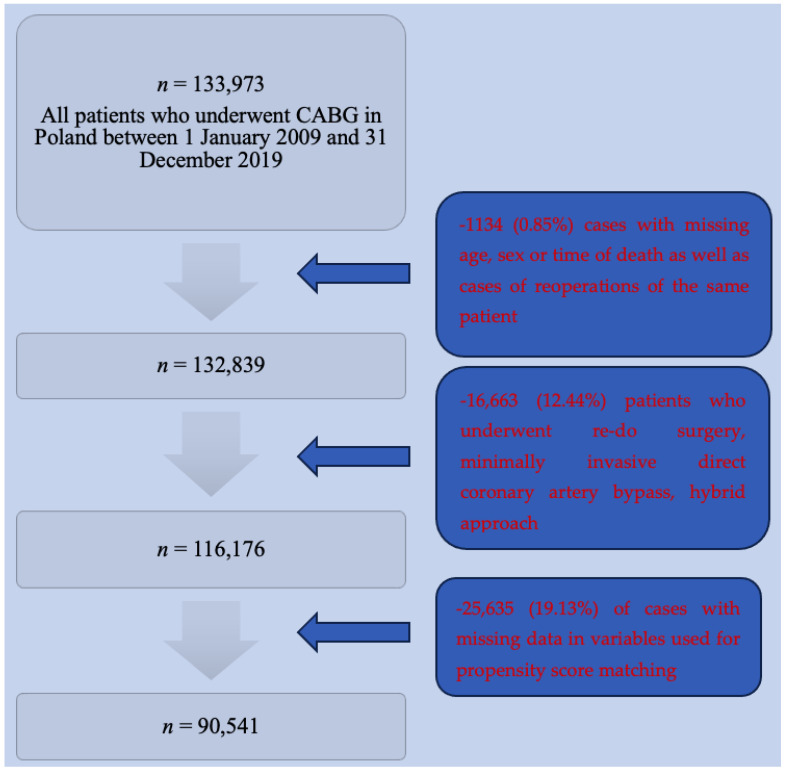
Patients included in the study.

The data from all 133,973 patients who underwent CABG procedure in Poland between 1 January 2009 and 31 December 2019 were included in the KROK Registry. Patients who underwent the same surgery for a second time, those who underwent minimally invasive direct coronary artery bypass, hybrid approach (17,797 patients, 13.3%), and patients for whom we lacked the data necessary to perform the matching procedure (25,635 patients, 19.1%) were excluded from the study (Figure 1).

The 30-day, annual, and long-term (13-year) mortality rates in groups of women and men were assessed. The mortality within the first year after surgery, considering age groups <60 years, 60–70 years, and >70 years, was compared.

The selection and exclusion of specific patient groups aimed to ensure data consistency and accuracy when assessing surgical risk and long-term outcomes.

### 1.5. Statistical Analysis

Continuous variables were presented as mean and standard deviation (when non-parametric tests were used for comparison, median values were also used), while categorical variables were presented as percentages. t-Student, Mann–Whitney-U, and Chi-squared tests were used to assess for statistical significance where appropriate.

Female and male patients were matched for comparison. The primary objective of the data matching process was to establish pairs of males and females that shared similar preoperative statuses. The degree of similarity was gauged through propensity scores, derived from logistic regression. The logistic regression model encompassed pertinent variables from Table 1, factors that might potentially influence treatment outcomes. The matching procedure utilized the greedy nearest neighbor algorithm, progressively pairing cases from both gender groups with the aim of minimizing within-pair distances while adhering to predetermined caliper values. The caliper radius, a critical parameter, stipulated the maximum acceptable disparity in propensity scores within each matched pair, preventing overly close matches that could compromise potential matches. The selected caliper radius value strikes a balance between forfeiting promising matches with overly restrictive values and compromising matching quality with excessively broad values. To this end, a caliper radius value of 0.2 times the pooled standard deviation was adopted in alignment with recommendations in the literature. According to simulations, a caliper radius of 0.2*Sigma was determined to eliminate 98% or more of bias in the crude estimator, generating confidence intervals with approximately accurate coverage rates [12]. The Mahalanobis distance metric was utilized to evaluate the similarities of the propensity scores between male and female subjects.

To evaluate the effectiveness of the matching process in achieving covariate balance, z-difference coefficients were calculated for each variable both before and after matching. The z-difference coefficients quantify the standardized differences in means or proportions between treated and control groups for each covariate. In this study, the mean z-difference coefficient before matching was −2.27, indicating significant imbalances between the groups. After the matching procedure was applied, the mean z-difference coefficient improved to −0.08, demonstrating a substantial reduction in covariate imbalances. Additionally, the variance of z-difference coefficients decreased from 197.17 before matching to 0.56 after matching. These results collectively suggest that the matching algorithm successfully mitigated covariate imbalances between the treatment groups, enhancing the comparability of the matched pairs. The analysis of all-cause mortality was included in the assessment of long-term follow-up data. All patients included in this study from the date of their procedure until 31 May 2022 were searched in the National Health Fund death database. These data were then analyzed using the Kaplan–Meier method with stratified log-rank testing. The date of operation was considered the starting point. To assess the presence of a trend in the proportion of operated women in successive years, the Chi-squared test for linear trends was used. To evaluate the trend in the number of surgeries performed, a generalized linear model with negative binomial distribution was used.

For analyses, a two-tailed *p*-value < 0.05 was considered statistically significant. The analyses and graphs were performed with the use of statistical software R version 4.2.1 2022 [13]. The matching procedure was carried out using the MatchIt R package [14]. Estimations of hazard functions were obtained with muhaz R package.

### 1.6. Results

The study group consisted of 90,541 patients 68,401 men (75.5%) and 22,140 women (24.5%). After propensity score matching, 22,117 men and women from each subgroup were obtained.

Detailed data from the KROK Registry enabled the assessment of patients in the following domains: baseline demographic data, individual risk factors, circulatory function, general condition before the surgery, procedure-related variables, and postoperative course variables, as well as quantitative variables, which are presented in Table 1, Table 2 and Table 3.

## 2. Mortality

**Figure 2:**
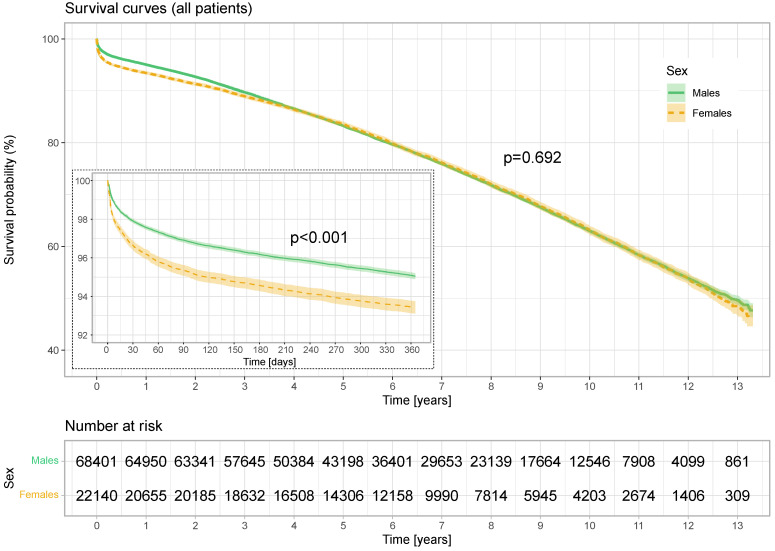
Annual and long-term survival (all patients). Kaplan–Meier survival curves of men and women for all-cause mortality. *p*: log-rank test.

**Figure 3:**
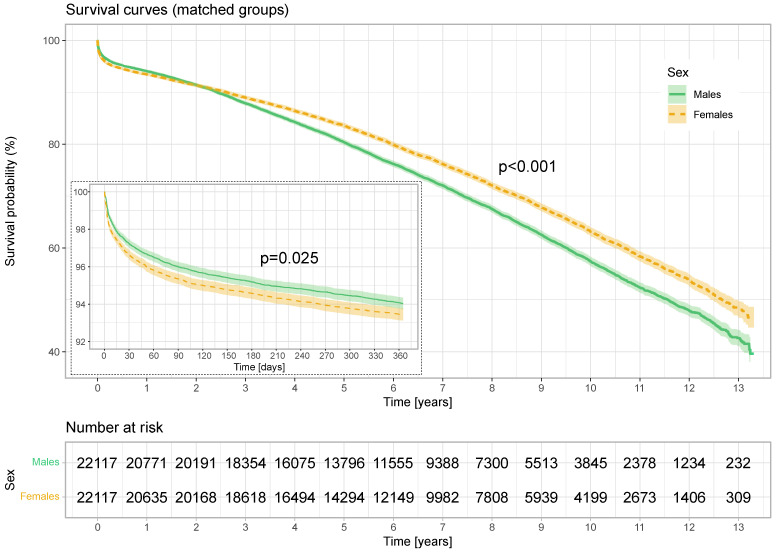
Annual and long-term survival (matched patients). Kaplan–Meier survival curves of men and women for all-cause mortality. *p*: log-rank test.

Kaplan–Meier survival curves of men and women operated on for coronary artery disease for all-cause mortality in 1-year follow-up and long-term follow-up of all patients and propensity-matched patients are presented in Figure 2 and Figure 3.

### 2.1. Comparison of Unmatched Groups

The rate of early mortality (30 days) in the male group was 2.1%, while in the female group, it was 3.4% (*p* < 0.001). The annual mortality rates were 4.9% vs. 6.6%, respectively (*p* < 0.001), and the long-term mortality rates were 28.2% vs. 28.8% (*p* = 0.692) (Table 4).

In the age group <60, in the annual observation, the mortality rate was lower among men than in women 2.17% vs. 2.93% (*p* = 0.005). In the 60–70 age group, the difference was even greater: 3.89% vs. 4.88% (*p* < 0.001). In the age group >70, the differences equalized 9.18% vs. 9.25% (*p* = 0.754).

Looking at the survival probability curves after the surgery in the unmatched population, in the first year of observation, there was a decrease in the survival rate in the women’s group (*p* < 0.001). This trend gradually decreased, and after 4 years the curves coincided and survival became stable, until the 10th year after surgery when better survival began to prevail again in men during the next follow-up period (*p* = 0.692).

### 2.2. Comparison of Propensity-Matched Groups

After propensity score matching, early mortality (30 days) was still significantly lower in the male group 2.8%, while in the female group it was 3.4% (*p* < 0.001). Similarly to those before adjustment, the annual mortality rates were 6.0% in men vs. 6.6% in women, respectively (*p* < 0.025). However, long-term mortality was higher in the male 33.0% vs. 28.8% in female group (*p* < 0.001) (Table 4).

In the age group <60, the annual mortality rate was lower among men (2.01%) than among women (2.93%) (*p* = 0.011). In the 60–70 age group, the difference was greater: 3.85% vs. 4.88%, respectively (*p* < 0.001). In the age group >70, the differences equalized: 9.35% vs. 9.24% (*p* = 0.845).

Looking at the survival probability curves in the matched population, the survival rate after the operation in the first year of observation was still lower in the female group (*p* = 0.025). However, after two years of observation, the survival probability curves crossed, and women showed better survival in the long term (*p* < 0.001).

## 3. Discussion

In the risk scales of perioperative death, female sex is recognized as one of the main independent risk factors [7,8,9]. However, there are other factors which may affect early and long-term outcomes that usually differ significantly to the disadvantages experienced by women [10,15,16]. The most important one among them is age. It is the strongest predictor of operative risk and is usually higher in women [10,15,16].

### 3.1. Unmatched Population Data

In the unmatched population, the average age of men was significantly lower than that of women, with the difference in median age reaching 4 years. We found that women were significantly more burdened with all traditional risk factors. However, left main (LM) stam stenosis and triple-vessel disease were significantly more frequent in men (*p* < 0.001).

In a recent meta-analysis, Gaudino et al. showed that only diabetes and obesity differentiated the female population, while hypertension distinguished men from women [1]. Some authors observed greater severity of coronary artery disease in women, but better ejection fraction [16]. Nuru et al. did not find greater burdens in the female group except for age and peripheral artery disease [17]. The fact that atherosclerosis affects women at older age, and it is often multivessel disease, changes patients’ risk profile [18].

Women had a longer hospital stay, higher incidence of respiratory complications, sternal wound infection, perioperative myocardial infarction, mechanical circulatory support, and neurological complications. This is consistent with data from the largest STS (the Society of Thoracic Surgeons) registry focused on neurological complications [19].

### 3.2. Matched Population Data

To address the potential confounding factors, we performed propensity-score matching. Before the matching, the men’s group had better outcomes for most of the preoperative parameters compared to the women’s group. Thus, the survival curves show a comparison of survival after CABG of younger men in better overall condition with a population of older and more burdened women. Due to the large difference in the numbers of patients in both groups, it was possible to select the appropriate number of men in terms of initial parameters for almost the same number of women as before matching.

Following the propensity-matching procedure, in patients’ baseline demographics, all preoperative differences between variables became non-significant. Only the EuroSCORE II (the surgical risk scoring tool) value remained higher in the women’s group. No differences were found between women and men regarding the surgical technique and any other procedure-related variables. This is an important finding meaning that gender did not determine the type and quality of the performed procedure.

Most postoperative complications occurred with similar frequency in both groups. Predisposition to wound infection exhibited a higher prevalence among females. Some investigators suggest that female sex constitutes a risk factor for sternal or leg wound infection after saphenous vein harvesting [20,21]. Furthermore, it is noteworthy that women with diabetes often exhibit suboptimal glycemic control, a well-established predisposing factor for wound infections [22].

Perioperative MI occurred more frequently in women. This may be explained by the smaller diameter of the native arteries and the greater tendency toward vascular spasm in the group of women [1].

The causes for the elevated frequency of reoperations due to bleeding within the males remain challenging to elucidate. Existing data do not establish a connection between gender and an increased postoperative bleeding risk [1,23]. One potential explanation could be linked to medications administered in the preoperative phase. Regrettably, The KROK registry does not include data concerning the use of antiplatelet drugs.

### 3.3. Mortality

In an unmatched population, mortality was significantly higher in the female group in short- and mid-term observations (*p* < 0.001). Better survival in the male group lasted for up to 3 years after surgery. Over the next 8 years, the survival rates remained similar, and in the last 2 years, there was a gradual trend towards improved survival in men, although it did not reach statistical significance. By evaluating mortality rates within three age groups, it was found that annual mortality in two younger age groups (both matched and unmatched) was higher in women, while in the oldest age group it was the same for both genders.

Several observational studies reported higher 30-day mortality rates in women [16,18,19]. A large meta-analysis from 2013, which included 966,492 CABG patients, demonstrated significantly higher mortality rates among women both in early and long-term observations during a 5-year period, both in the overall group and in the matched group [4]. Some recent studies confirm this observation [2,10]. Only a few studies indicate increased postoperative mortality in women but show the equalization of mortality rates between genders in later observations [19].

All publications assessing mortality in specific age groups consistently report significantly increased mortality in the youngest group of post-operation women, while in the oldest group, mortality is similar or even lower than in men [1,2,16]. In 2002, in the largest registry-based study to date, which included 51,187 patients, Vaccarino et al. assessed differences in early mortality between women and men after CABG. The authors found significantly increased mortality rates among women after surgery in all age groups, with the greatest difference observed in the younger patient group [16]. In the presented study, in both younger age groups, regardless of whether the groups were matched or not, the mortality rate for men was lower than for women; however, the most pronounced difference was observed in the middle-aged group. In the most advanced age group, women and men had an equal frequency of mortality. This is somewhat contradictory to previously cited studies in which the highest mortality rate was observed in the youngest group of women. This is also contradictory to the reported older age of operated women, which according to risk scores, would be a cause of increased female mortality [7,8,9,19]. This issue requires a separate analysis and perhaps further research.

After matching, early and mid-term mortality remained higher among women, but after 2 years, male mortality exceeded female mortality and increased throughout the observation period. This probably means that although the increased surgical risk in women provides a survival advantage for men in the early postoperative period, men have a shorter lifespan in the long-term observation, which is likely related to the global trend of higher mortality rates among men [24]. This result does not have its equivalent in older studies. A recent review of Gaudino et al. showed that women have similar outcomes in 5-year observations [1]. Nuru et al. also observed increased early and mid-term mortality and better long-term survival among women [17]. This is the only publication consistent with our observations. The similarity between these studies lies in the fact that both are European registries and cover a similar subject, but they greatly differ in the size of the study groups, as the Norwegian study is a single-center study. In the recent literature, only Abreau et al. has demonstrated, on adjusted male and female populations, that long-term results are similar, and gender is not an independent risk factor of mortality and should not influence decision-making regarding revascularization strategy [10].

A limitation of this study lies in its exclusive reliance on data transferred from the medical registry, leading to a retrospective design. Analyzing extensive data from such sources results in inherent challenges like selection bias and incomplete data. It is important to mention that the data integrated into the KROK Registry exhibit heterogeneity. Additionally, there were 22,140 female participants (24.5%), and 68,401 male patients (75.55%). Furthermore, the current investigation hinged on registry data that were strictly confined to the information available within the KROK database, which does not contain information about postprocedural pharmacological treatment, laboratory investigations along with new diseases diagnosed, or detailed echocardiographic findings [25]. There was no further information about the quality of life or functional status of the patients. The follow-up analysis was limited to all-cause mortality. Because of this, it is impossible to assess whether the patient’s death after discharge was related to the CABG procedure or was due to unrelated factors [26].

## 4. Conclusions

It was found that women undergoing CABG had worse preoperative profiles than men. Initially higher surgical risk resulted in higher early mortality in women. In matched patients, early mortality was also lower for men, but the long-term survival becomes better in women. Gender should not be a discriminating factor in determining the surgical strategy.

## Figures and Tables

**Table 1:** Comparison of preoperative variables in the whole group (left), and propensity-matched patients (right).

Preoperative Status Variables							
		All Patients (*n* = 90,541)			Matched Patients (*n* = 44,234)		
Group of Variables	Variable	Men (*n* = 68,401)	Women (*n* = 22,140)	*p* Value	Men (*n* = 22,117)	Women (*n* = 22,117)	*p* Value
Demographic data	Age, y	65.08 ± 8.67	68.44 ± 8.23	0.001	68.42 ± 8.50	68.43 ± 8.23	0.956
Circulatory function	CCS IV, *n* (%)	6130 (9)	2446 (11)	0.001	2422 (11)	2435 (11)	0.843
	NYHA III-IV, *n* (%)	7717 (11.3)	2879 (13)	0.001	2807 (12.7)	2873 (13)	0.348
	Recent MI, *n* (%)	21,920 (32)	7189 (32.5)	0.240	7112 (32.3)	7180 (32.5)	0.489
	LVEF < 30%, *n* (%)	2278 (3.3)	321 (1.4)	0.001	336 (1.5)	321 (1.5)	0.555
	Chronic AF, *n* (%)	3558 (5.2)	1028 (4.6)	0.001	1025 (4.6)	1027 (4.6)	0.964
	Left main stem lesion, *n* (%)	21,725 (31.8)	5970 (27)	0.001	5914 (29.7)	5968 (27)	0.562
	Three-vessel disease, *n* (%)	43,354 (63.4)	13,324 (60.2)	0.001	13,239 (59.9)	13,316 (60.2)	0.455
Individual risk factors	Smoking, *n* (%)	14,260 (20.8)	2831 (12.8)	0.001	2882 (13.0)	2831 (12.8)	0.470
	Hypercholesterolemia, *n* (%)	46,181 (67.5)	15,251 (68.9)	0.001	15,220 (68.8)	15,236 (68.9)	0.870
	Diabetes, *n* (%)	23,815 (34.8)	9689 (43.8)	0.001	9684 (43.8)	9671 (43.7)	0.901
	Arterial hypertension, *n* (%)	60,451 (88.4)	20,427 (92.3)	0.001	20,377 (92.1)	20,404 (92.3)	0.632
	BMI > 35, kg/m^2^, *n* (%)	4183 (6.1)	2302 (10.4)	0.001	2256 (10.2)	2282 (10.3)	0.684
	Renal failure, *n* (%)	4421 (6.5)	1586 (7.2)	0.001	1613 (7.3)	1582 (7.2)	0.569
	COPD, *n* (%)	5040 (7.4)	1422 (6.4)	0.001	1460 (6.6)	1422 (6.4)	0.464
	Past TIA, RIND, *n* (%)	1405 (2.1)	491 (2.2)	0.139	453 (2)	490 (2.2)	0.223
	Past CAS, *n* (%)	396 (0.6)	116 (0.5)	0.343	111 (0.5)	116 (0.5)	0.739
	PVD, *n* (%)	10,965 (16)	3441 (15.5)	0.084	3445 (15.6)	3437 (15.5)	0.916
Condition before the procedure	Cardiogenic shock, *n* (%)	313 (0.5)	124 (0.6)	0.056	119 (0.5)	123 (0.6)	0.797
	Use of IABP, *n* (%)	601 (0.9)	217 (1)	0.165	219 (1)	216 (1)	0.885
	i.v. nitrates or heparin, *n* (%)	7277 (10.6)	2517 (11.4)	0.002	2498 (11.3)	2509 (11.3)	0.869

Abbreviations: AF—atrial fibrillation, CAS—carotid artery stenting, CCS—Canadian Coronary Score, COPD—chronic obstructive pulmonary disease, IABP—intra-aortic balloon pump, i.v.—intra venous, LVEF—left ventricular ejection fraction, MI—myocardial infarction, NYHA—New York Heart Association, PVD—peripheral vascular disease, RIND—reversible ischemic neurologic deficit, TIA—transient ischemic attack.

**Table 2:** Comparison of quantitative characteristics between women and men in matched and non-matched groups.

			All Patients (*n* = 90,541)					Matched Patients (*n* = 44,234)				
			*n*	Mean	SD	Median	*p* Value	*n*	Mean	SD	Median	*p* Value
Preoperative variables	Age, y	Women	22,140	68.440	8.235	68.871	0.001	22,117	68.427	8.229	68.858	0.956
		Men	68,401	65.082	8.675	64.815		22,117	68.423	8.502	68.587	
		All	90,541	65.903	8.690	65.811		44,234	68.425	8.367	68.734	
	EuroSCORE II (available since 2012)	Women	16,436	2.770	3.676	1.830	0.001	16,420	2.763	3.643	1.827	0.001
		Men	51,558	1.905	2.594	1.252		16,462	2.159	2.940	1.415	
		All	67,994	2.114	2.917	1.371		32,882	2.461	3.324	1.608	
Operating variables	Operating time, hours	Women	22,118	3.336	1.333	3.167	0.001	22,095	3.337	1.333	3.167	0.053
		Men	68,329	3.408	1.338	3.250		22,093	3.361	1.347	3.250	
		All	90,447	3.390	1.337	3.250		44,188	3.349	1.340	3.250	
	Anastomosis number	Women	22,118	2.715	1.157	3.000	0.001	22,095	2.716	1.157	3.000	0.200
		Men	68,349	2.839	1.186	3.000		22,104	2.731	1.193	3.000	
		All	90,467	2.809	1.180	3.000		44,199	2.724	1.175	3.000	
	Follow-up time, y	Women	22,140	6.568	3.425	6.507	0.017	22,117	6.569	3.424	6.507	0.001
		Men	68,401	6.505	3.341	6.315		22,117	6.390	3.366	6.217	
		All	90,541	6.521	3.361	6.365		44,234	6.480	3.396	6.362	
Postoperative variables	Hospital stay, days	Women	21,992	10.946	7.361	9.000	0.001	21,969	10.943	7.356	9.000	0.001
		Men	67,943	10.378	6.523	9.000		21,958	10.607	6.738	9.000	
		All	89,935	10.517	6.742	9.000		43,927	10.775	7.056	9.000	
	Ventilation * time, hours	Women	18,056	20.321	124.1	8.333	0.484	18,035	20.319	124.2	8.333	0.301
		Men	56,138	19.574	126.7	7.583		18,113	21.798	146.6	7.917	
		All	74,194	19.756	126.1	7.833		36,148	21.060	135.9	8.083	

* Ventilation: —unmatched: the percent of patients with postoperative ventilation exceeding 24 h was significantly higher in female patients (7.2% versus 6.5%, *p* = 0.006). —matched: the percentage of patients with postoperative ventilation exceeding 24 h did not differ between the groups (7.2% versus 7.2%, *p* = 0.944).

**Table 3:** Comparison of procedure-related variables (upper part) and postoperative complications (lower part) in all patients (left) and in propensity-matched patients (right).

	All Patients (*n* = 90,541)			Matched Patients (*n* = 44,234)		
	Men (*n* = 68,401)	Women (*n* = 22,140)	*p* Value	Men (*n* = 22,117)	Women (*n* = 22,117)	*p* Value
Procedure related variables						
Non-elective surgery, *n* (%)	25,471 (37.2)	8597 (38.8)	0.001	8547 (38.6)	8583 (38.8)	0.725
Complete arterial revascularization, *n* (%)	10,848 (15.9)	3480 (15.7)	0.617	3544 (16.0)	3472 (15.7)	0.349
1 graft, *n* (%)	6439 (9.4)	2612 (11.8)	0.001	2619 (11.8)	2596 (11.7)	0.735
2 grafts, *n* (%)	25,513 (37.3)	8910 (40.2)	0.001	9003 (40.7)	8904 (40.3)	0.338
3 or more grafts, *n* (%)	36,429 (53.3)	10,609 (47.9)	0.001	10,486 (47.4)	10,608 (48)	0.245
On-pump CABG, *n* (%)	41,500 (60.7)	13,006 (58.7)	0.001	12,811 (57.9)	12,999 (58.8)	0.07
Off-pump CABG, *n* (%)	26,901 (39.3)	9134 (41.3)	0.001	9306 (42.1)	9118 (41.2)	0.07
Postoperative complications						
Neurological complications, *n* (%)	922 (1.35)	370 (1.67)	0.001	330 (1.49)	370 (1.67)	0.137
Respiratory complications, *n* (%)	2034 (2.97)	772 (3.49)	0.001	788 (3.56)	768 (3.47)	0.624
Gastrointestinal complications, *n* (%)	430 (0.63)	161 (0.73)	0.125	159 (0.72)	161 (0.73)	0.955
Renal complications, *n* (%)	838 (1.23)	299 (1.35)	0.155	334 (1.51)	298 (1.35)	0.161
Sternal, mediastinal or wound infection, *n* (%)	1338 (1.96)	517 (2.34)	0.001	437 (1.98)	516 (2.33)	0.011
Perioperative myocardial infarction, *n* (%)	747 (6.51)	369 (9.42)	0.001	251 (6.95)	368 (9.41)	0.001
Mechanical circulatory support, *n* (%)	354 (0.52)	186 (0.84)	0.001	106 (0.48)	185 (0.84)	0.001
ICU readmission, *n* (%)	552 (0.81)	200 (0.90)	0.183	211 (0.95)	199 (0.90)	0.585
Reoperation due to bleeding, *n* (%)	2811 (4.11)	714 (3.22)	0.001	948 (4.29)	713 (3.22)	0.001

Abbreviations: CABG—coronary artery bypass grafting, ICU—intensive care unit.

**Table 4:** Number of events (deaths) in both unmatched and matched patient’s groups.

		Females	Males	Total
All patients (*n* = 90,541)	30-days mortality	745 (3.36%)	1433 (2.09%)	2178 (2.41%)
	1-year mortality	1452 (6.56%)	3385 (4.95%)	4837 (5.34%)
	Total mortality	6377 (28.80%)	19,298 (28.21%)	25,675 (28.36%)
Matched patients (*n* = 44,234)	30-day mortality	743 (3.40%)	611 (2.80%)	1354 (3.10%)
	1-year mortality	1449 (6.60%)	1320 (6.00%)	2769 (6.30%)
	Total mortality	6365 (28.80%)	7307 (33.00%)	13,672 (30.90%)

## Data Availability

The raw data supporting the conclusions of this article will be made available by the authors on reasonable request.
